# Outcomes of dose-adjusted Berlin–Frankfurt–Münster-90 regimen without radiotherapy in adolescents and adults with T cell lymphoblastic lymphoma

**DOI:** 10.1007/s12032-015-0551-9

**Published:** 2015-03-10

**Authors:** Yan Xie, Yuntao Zhang, Wen Zheng, Xiaopei Wang, Ningjing Lin, Meifeng Tu, Lingyan Ping, Zhitao Ying, Chen Zhang, Weiping Liu, Lijuan Deng, Yuqin Song, Jun Zhu

**Affiliations:** Key Laboratory of Carcinogenesis and Translational Research (Ministry of Education), Department of Lymphoma, Peking University Cancer Hospital & Institute, Beijing, People’s Republic of China

**Keywords:** T cell lymphoblastic lymphoma, Adolescents, Adults, Dose-adjusted BFM-90 regimen, Prognosis

## Abstract

The aim of this study was to evaluate the outcomes using the dose-adjusted Berlin–Frankfurt–Munster (BFM-90) regimen without radiotherapy in adolescents and adults with T cell lymphoblastic lymphoma (T-LBL) at Beijing Cancer Hospital. Between March 2004 and December 2013, 57 newly diagnosed T-LBL patients were treated in our center. We retrospectively analyzed their main clinical characteristics and prognosis. The media age of the patients at diagnosis was 26 (range 14–54). At a median follow-up of 24 months (range 5–119), 38 patients (67 %) were alive. The estimated 3-year overall survival (OS) rate and progression-free survival (PFS) rate were 64 and 60 %, respectively. Abnormal WBC at diagnosis, high IPI and no early response were indicated as adverse prognostic factors for both PFS and OS (*p* < 0.05). There was also a trend for better survival in autologous peripheral blood stem cell transplantation (APBSCT) group as compared to non-APBSCT group (3-year OS 83 vs. 57 %), but without any significant difference. This study suggested that the dose-adjusted BFM-90 protocol without irradiation showed comparable long-term results in Chinese adolescents and adults with T-LBL. APBSCT may become a choice whether we can identify the best candidate.

## Introduction

T cell lymphoblastic lymphoma (T-LBL) and T cell acute lymphoblastic leukemia (T-ALL) are categorized as precursor T cell malignancy, frequently accompanied by a mediastinal mass and a high prevalence of central nervous system (CNS) involvement [1]. Because T-LBL cell marker expression overlaps that of T-ALL, the clinical distinction between the two entities is arbitrarily determined by the degree of bone marrow (BM) involvement: Patients with more than 25 % lymphoblasts are classified as having T-ALL, whereas those with a lesser degree of marrow replacement are classified as having T-LBL [2]. T cell lymphoblastic lymphoma/leukemia accounts for approximately 3.4 % of all non-Hodgkin lymphomas (NHLs) in China [3]. The conventional CHOP-like regimen produced low complete remission (CR) rate(17 %) and short media overall survival (OS) (8.5 months) [4]. But the prognosis of LBL has dramatically improved with the use of intensive ALL-type chemotherapy regimens, with an event-free survival (EFS) of 90 % in children and disease-free survival (DFS) of 72 % in adults [5, 6]. Although the use of ALL-type regimens such as BFM-90 regimen usually acquired better survival rates, they brought more adverse events as well as treatment-related death. The aim of this study was to evaluate the outcomes using the dose-adjusted Berlin–Frankfurt–Munster (BFM-90) regimen without radiotherapy in adolescents and adults in our cancer center.

## Materials and methods

### Patients

Between March 2004 and December 2013, patients not less than 14 years old with untreated T-LBL were retrospectively analyzed. All cases were pathologically diagnosed by biopsy material from a lymph node or tumor mass according to WHO classification of hematological malignancies in Beijing Cancer Hospital. A series of markers, including CD1a, CD3, CD4, CD8, CD10, CD20, CD45RO, CD79a, Ki-67 and terminal deoxynucleotidyl transferase (TdT), were performed by immunohistochemistry.

A complete panel was performed at the time of baseline staging, re-staging and final evaluation. This panel included a full clinical history, physical examination, complete hematological and biochemical tests, computed tomography (CT) scans of neck, chest, abdomen and pelvic, or 18F-fluorodeoxyglucose positron emission tomography (18 F-FDG-PET) if possible, cerebrospinal fluid examination, BM aspirate and/or biopsy. Staging was carried out according to the Ann Arbor system for NHL. Diagnosis of central nervous system (CNS) disease was made in case of blast cells found in the cerebrospinal fluid (CSF) or cerebral infiltration on cranial CT. It is important to note that patients who had more than 25 % blasts in the BM but presented as a bulky tumor were also included in this study.

### Treatment

All patients received a dose-adjusted BFM-90 regimen after they or their guardians signed the informed consent. The protocol was derived from BFM-90 regimen using the same major drugs (prednisone, vincristine, asparaginase, cyclophosphamide, cytarabine, daunorubicin, doxorubicin, methotrexate and 6-mercaptopurine), but the doses and frequency of methotrexate and cytarabine were reduced. Cranial prophylactic radiotherapy was omitted, but all patients received regular intrathecal chemotherapy. Local irradiation was also omitted in this study. The protocols for induction, consolidation, re-induction and maintenance are listed in Table 1. If the patient achieved complete remission (CR) or partial remission (PR) and agreed to receive high-dose chemotherapy plus autologous peripheral blood stem cell transplantation (APBSCT), it should be given after re-induction phase. After APBSCT, the patient would continue with maintenance therapy.

**Table 1:** Protocols of modified BFM-90

Drugs	Doses	Days
Induction phase 1a		
Prednisone (PDN)	60 mg/m^2^ oral	1–28, then taper over 3 × 3 days
Vincristine (VCR)	1.5 mg/m^2^ (max 2 mg) IV	8,15,22,29
Daunorubicin (DNR)	30 mg/m^2^ IV	8,15,22,29
L-asparaginase (L-ASP) Or Pegasparaginase	6,000 IU/m^2^ IV Or 2500 IU/m^2^ IM	8–15 Or 8,22
^a^IT chemotherapy		^b^1,15
Induction phase 1b		
Cyclophosphamide (CTX)	1000 mg/m^2^ IV	1,15
Cytarabine (Ara-C)	75 mg/m^2^ IV	3–6, 17–20
6-Mercaptopurine (6-MP)	60 mg/m^2^ oral	1–28
^a^IT chemotherapy		1,15
Consolidation phase		
6-Mercaptopurine (6-MP)	25 mg/m^2^ oral	1–28
^c^ Methotrexate (MTX)	3000 mg/m^2^ IV	1,15
^a^IT chemotherapy		1,15
Reinduction phase 2a		
Dexamethasone (DXM)	9 mg/m^2^ oral	1–21, then taper over 3 × 3 days
Vincristine (VCR)	1.5 mg/m^2^ (max 2 mg) IV	1,8,15,22
Daunorubicin (DNR)	30 mg/m^2^ IV	1,8,15,22
L-asparaginase (L-ASP) Or Pegaspargase	6,000 IU/m^2^ IV Or 2500 IU/m^2^ IM	1–8 Or 1,15
^a^IT chemotherapy		1,15
Reinduction phase 2b		
Cyclophosphamide (CTX)	1000 mg/m^2^ IV	1,15
Cytarabine (Ara-C)	75 mg/m^2^ IV	3–6, 17–20
6-Mercaptopurine (6-MP)	60 mg/m^2^ oral	1–28
^a^IT chemotherapy		1,15
Maintenance phase		
Methotrexate (MTX)	20 mg/m^2^ oral	Once a week for 12 months
6-Mercaptopurine (6-MP)	60 mg/m^2^ oral	Daily for 12 months

^a^Intrathecal (IT) chemotherapy with cytarabine (50 mg) and/or methotrexate (10 mg) and dexamethasone (5 mg)

^b^If the patient had CNS involvement at diagnosis, two doses IT would be added at day 8 and 22

^c^One-tenth of the methotrexate dose was administered within 0.5 h, and nine-tenths given by intravenous (IV) drip over 23.5 h, Citrovorum folinate rescue was given at a dose of 30 mg/m^2^ at 36 h and then 15 mg/m^2^ at 42, 48, 54, 60, 66, 72, 78 and 84 h

### Response evaluation and statistical analysis

Response was evaluated at the end of each phase of the protocol according to Cheson criteria. Overall survival (OS) was calculated from the date of diagnosis to the date of death by any cause or the last follow-up in survivors. Progression-free survival (PFS) was measured from the date of diagnosis to the first sign of progression, relapse after response, death from any cause or to the date of last follow-up. Patients who were lost to follow-up were censored at the time of their last follow-up examination. OS and PFS curves were estimated using the Kaplan–Meier method and were compared using the log-rank test. Prognostic factors affecting OS and PFS were assessed by the log-rank test. Statistically significant differences were defined as two-sided *p* values < 0.05. All statistical analyses were performed using SPSS 13.0 statistical software (SPSS, Chicago, IL).

## Results

### Patient characteristics

A total of 57 patients were consecutive newly diagnosed as T-LBL and treated with the dose-adjusted BFM-90 regimen in our study. Their characteristics are listed in Table 2. Their age ranged from 14 to 54 years, with a median age of 26 years; 72 % cases were male. The majority of patients presented with stage III or IV disease (47/57, 82 %). BM infiltration was detected in 33 patients (58 %), 16 (28 %) of whom had more than 25 % blast cells in the BM. Four (7 %) cases were diagnosed as initial CNS invasion, including two patients with concurrent CNS and BM involvement. Twenty-two (39 %) patients presented with bulky mass (>7.5 cm), and 23 (40 %) patients had LDH levels higher than the institutional upper limit of normal (ULN). White blood cells (WBC) were found to be abnormal in 24 (42 %) patients at diagnosis. Thirty-four (60 %) patients were within international prognostic index (IPI) group 0–1, while 23 were within IPI group 2–4. Thirty-four (69 %) patients had a Ki-67 score ≥75 % (data unavailable in eight patients).

**Table 2:** Patients’ characteristics and prognostic factors for overall survival (OS) and progression-free survival (PFS)

Variable	Number (%)	3-year OS (%)	*p* value	3-year PFS (%)	*p* value
Gender					
Male	41 (72 %)	63		58	
Female	16 (28 %)	69	0.965	70	0.879
Stage					
I + II	10 (18 %)	80		88	
III + IV	47 (82 %)	60	0.138	53	0.085
Ki-67					
≥75 %	34 (69 %)	71		67	
<75 %	15 (31 %)	60	0.283	52	0.518
LDH					
>ULN	23 (40 %)	47		40	
≤ULN	34 (60 %)	76	0.154	72	0.023
WBC					
Normal	33 (58 %)	79		76	
Abnormal	24 (42 %)	44	0.033	37	0.031
CNS involvement					
Yes	4 (7 %)	50		25	
No	53 (93 %)	67	0.351	65	0.180
BM involvement					
Yes	33 (58 %)	57		42	
No	24 (42 %)	72	0.130	76	0.060
Bulky mass(> 7.5 cm)					
Yes	22 (39 %)	65		67	
No	35 (61 %)	64	0.731	56	0.645
IPI					
0–1	34 (60 %)	80		80	
2–4	23 (40 %)	45	0.027	34	0.004
Response					
CR	43 (75 %)	74		69	
PR	11 (19 %)	41		23	
PD	3 (5 %)	0	0.000	0	0.000
APBSCT					
Yes	17 (30 %)	83		75	
No	40 (70 %)	57	0.133	54	0.064

### Therapeutic results and survival

Forty-three (75 %) patients achieved CR and 11 (19 %) achieved PR after induction phase. Among those who achieved CR, 16 patients proceeded to APBSCT. One patient who only achieved PR also received APBSCT after re-induction phase. Three patients had progression disease after induction treatment. Two of them died at 6 and 8 months, respectively. The other patient received a salvage therapy and was still alive at his last follow-up visit.

**Fig. 1:**
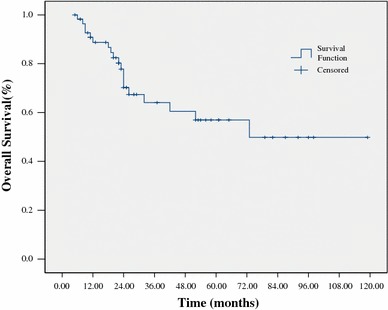
Overall survival for all patients with T-LBL

**Fig. 2:**
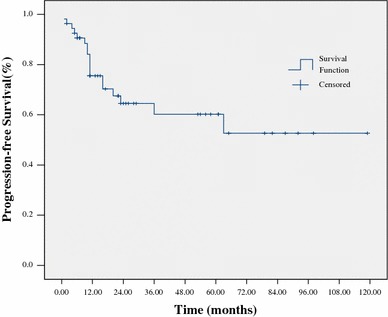
Progression-free survival for all patients with T-LBL

**Fig. 3:**
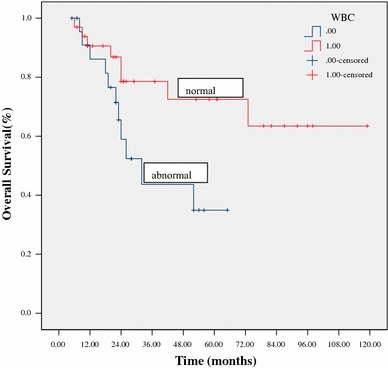
Overall survival stratified by white blood cell counts at diagnosis

**Fig. 4:**
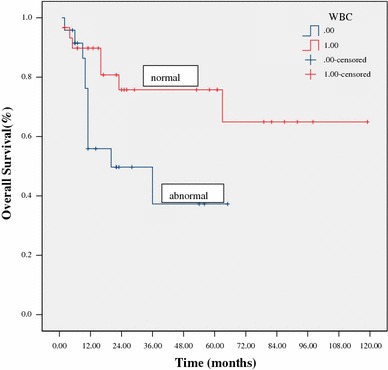
Progression-free survival stratified by white blood cell counts at diagnosis

**Fig. 5:**
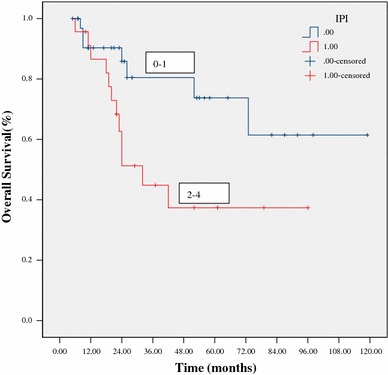
Overall survival stratified by IPI score at diagnosis

**Fig. 6:**
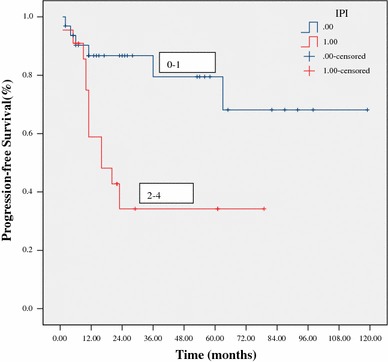
Progression-free survival stratified by IPI score at diagnosis

At a median follow-up of 24 months (range 5–119) for all patients, 38 patients (67 %) were alive. The estimated 3-year OS and PFS were 64 % and 60 %, respectively (Figs. 1, 2). The 3-year OS and PFS were significantly better in patients with normal WBC counts at diagnosis as compared to those with abnormal WBC counts (79 vs. 44 %, *p* = 0.033; 76 vs. 31 %, *p* = 0.031, respectively) (Figs. 3, 4). The 3-year OS and PFS probability was significantly worse in IPI group 2–4 when compared to IPI group 0–1 (45 vs. 74 %, *p* = 0.027; 34 vs. 80 %, *p* = 0.004, respectively) (Figs. 5, 6). Patients with elevated LDH at diagnosis were significantly associated with poor 3-year PFS (40 %, *p* = 0.023), but there was no statistically difference in the 3-year OS (*p* = 0.154). There was a significant difference between 3-year OS and PFS by treatment response. A trend of better survival was shown in APBSCT group as compared to non-APBSCT group (3-year OS: 83 vs. 57 %; 3-year PFS: 75 vs. 54 %); however, the result did not show significant difference. There was no statistically difference in 3-year OS or PFS according to gender, stage, Ki-67, CNS involvement, BM involvement or bulky mass (Table 2).

Twenty-five patients experienced progression or relapse. Among them, 23 cases progressed or relapsed during the first 2 years and the other two cases relapsed at 36 and 68 months, respectively. Sites of relapse included lymph node (*n* = 17), BM (*n* = 6) and CNS (*n* = 2). All patients with BM relapse had BM involvement at diagnosis, but only one patient with CNS relapse had CNS invasion at diagnosis. All of the patients with BM or CNS relapse died. Seven patients with lymph node relapse were alive at the last visit. Five cases were still in salvage therapy, but only one of them achieved PR. One patient accepted allogeneic stem cell transplant and remained in CR at 52 months of follow-up. One patient only accepted salvage chemotherapy and showed no sign of progression at 96 months.

### Toxicity

No death occurred due to toxicity. The most common grade 3 and 4 adverse events were hematologic events, including neutropenia (in 86 % cases), thrombocytopenia (in 56 %), anemia (in 49 %) and febrile neutropenia (in 46 %). Grade 3 aminotransferase elevation was seen in six patients. One patient developed acute renal failure due to high-dose methotrexate, and one patient suffered from acute pancreatitis due to pegylated asparaginase. No other grade 3 or 4 non-hematologic adverse events occurred.

## Discussion

T cell lymphoblastic lymphoma (T-LBL) is an uncommon, aggressive but curable T cell lymphoma in adult, but more common in younger adults [7]. In our study, the patients showed clinical characteristics as published data: younger age, predominantly male, advanced stage and higher BM involvement.

In our study, we tried the dose-adjusted BFM-90 regimen and observed a CR rate of 75 % and a PR rate of 19 % at the end of induction phase, and the 3-year PFS and OS were 60 and 64 %, respectively. Despite the lower CR rate, we achieved a comparable overall response rate (ORR 94 %) and long-term outcome, comparing favorably with those obtained in Thomas et al. study and Wang K et al. study [8, 9]. The most common grade 3 and 4 adverse events still were hematologic events, but overall tolerance was acceptable with no treatment-related mortality. It showed that our dose-adjusted BFM-90 regimen had similar efficacy and acceptable toxicity.

Relapse and progression occurred in 25 of 57 patients in this study, and their outcomes were very poor. If we excluded the five alive patients on therapy, only two patients survived free of the disease in this study, supporting literature about the difficulty in salvaging patients with recurrent T-LBL [10–12]. More effective new drugs such as nelarabine and NOTCH-1 inhibitors are needed to improve the outcome of patients with relapsed disease. Most relapses occurred within the first 2 years, similar to Termuhlen et al. report [13]. But it should be noted that late relapse still happened up to 68 months. Lymph nodes (17/25) remained the most common site of relapse, but with disseminated sites, which suggested that local irradiation might not prevent relapse and systemic therapy was more important. There are no sufficient data to analyze the specific precise values of intrathecal therapy, systemic CNS-penetrating therapy and cranial irradiation as prophylaxis methods. Consecutive studies from the BFM group introduced four doses of intravenous methotrexate at 5 g/m^2^, and in NHL/BFM-95, prophylactic cranial irradiation was safely eliminated [5, 14]. The Children’s Cancer Group (CCG) improved the outcome in ALL patients using a modified BFM approach without high-dose methotrexate or cranial irradiation, and CNS prophylaxis was frequent delivery of intrathecal chemotherapy [13, 15]. In our study, we gave patients two doses of intravenous methotrexate at 3 g/m^2^ combined with 10–12 doses intrathecal chemotherapy without prophylactic cranial irradiation. Two patients (3.5 %, 2/57) had CNS relapse, and one of them had CNS invasion at diagnosis. The incidence of CNS relapse occurred in our group was comparable to that reported by Hoelzer et al. [6] and Möricke et al. [16], all less than 5 %. The low incidence of CNS relapse supports the efficacy of such CNS prophylaxis method, but it needs larger scale and prospective trials to confirm.

Song et al. [17] suggested consolidation with SCT in first response in chemosensitive T-LBL adult patients, which showed favorable long-term outcome [17]. Sweetenham et al. [18] conducted a prospective multicenter randomized trial to determine whether autologous stem cell transplantation (ASCT) was superior to conventional-dose consolidation and maintenance chemotherapy (CC) as post-remission therapy in LBL. With a median follow-up of 37 months, the actuarial 3-year relapse-free survival rate was 24 % for the CC arm and 55 % for the ASCT arm (*p* = 0.065). The OS rates were 45 and 56 %, respectively (*p* = 0.71). In our study, 17 patients proceeded with APBSCT followed by maintenance therapy. There was also a trend for better survival in APBSCT group compared to non-APBSCT group (3-year OS: 83 vs. 57 %); however, the result did not show significance difference. We need to increase the number of patients and identify the best candidate for APBSCT.

To date, reliable and reproducible prognostic factors for adult LBL have not been described [6, 9, 13, 19]. This study confirms the previously published findings that gender, stage, a bulky mass, elevated LDH, CNS or BM infiltration did not have a significant effect on OS. And in our series, abnormal WBC at diagnosis and high IPI were indicated as adverse prognostic factors for both EFS and OS, in contrast to report from Hoelzer et al. [6]. We also found that early response in such patient was an important prognostic factor. Patients who achieved CR or PR after induction therapy (8 weeks) acquired a better outcome. This suggests that we should adjust the treatment strategy as earlier as response evaluation is not satisfactory after induction regimen.

This study suggested that the dose-adjusted BFM-90 protocol without irradiation showed comparable long-term survival and control of CNS recurrence in Chinese adolescents and adults with T-LBL. Although APBSCT in this group did not improve survival, it may become a choice whether we can identify the best candidate.
